# From the Lectures of Prof. Davis in the Medical, and Prof. Andrews in the Surgical Wards of the Mercy Hospital

**Published:** 1867-05

**Authors:** 


### FROM THE LECTURES OF PROF. DAVIS IN THE MEDICAL, AND PROF. ANDREWS IN THE SURGICAL WARDS OF THE MERCY HOSPITAL.

[*Reported for the Examiner.*]

[The Lectures from which these "Notes" are made, being delivered before the Summer Class of the Chicago Medical College, necessarily contain much matter of a purely elementary character. No attempt is, therefore, made to report them in full, as such information, for the majority of the readers of the Examiner, would be, it is presumed, supererogatory.

The "Notes" are intended simply to comprise outlines of such cases as may possess sufficient importance, together with a rationale of the treatment or summary of operation, and such didactic hints as may seem valuable—aiming to be a practical elucidation of the treatment, medical and surgical, rather than a syllabus of the Lectures.—R.]

SERVICE PROF. N. S. DAVIS, M.D., VISITING PHYSICIAN.

*Therapeutic Value of Tinct. Chloride of Iron and of the Sulphites in Treatment of Erysipelas.*—Ætat. 41; American; occupation, carpenter; admitted for treatment, March 27th, laboring under a well-marked attack of *facial erysipelas*, the inflammatory stage having probably existed about forty-eight hours, although he had been complaining previously, and had taken some purgative medicine before his admission into the hospital. The febrile symptoms not being very urgent, and there being no evidence of cerebral congestion, he was ordered twenty-five drops of the tinct. chloride of iron (*tincture ferri chloridi*, Ph. U. S.), well diluted with sugar and water, every two hours during the ensuing twenty-four; after which, the interval was to be lengthened to three hours during the second day, and to four hours on the third day, when it was confidently anticipated the septic character of the disease would be entirely destroyed, and convalescence established.

The sulphites of soda and lime would undoubtedly produce the same results as antiseptics—in fact, their efficiency in the treatment of erysipelas, as well as in all other of the large and important class of catalytic diseases, seems to depend almost exclusively, on their power to *neutralize* the blood-poison. The tinct. chloride of iron possesses this power to a great extent; but in addition to this, it is one of the most reliable tonics, and where not contra-indicated by a high degree of febrile excitement, by cerebral congestion, by such constitutional disturbance as is manifested by a strong, full, and frequent pulse, excessive heat of surface, and severe pain, we will find its use followed by a prompter convalescence, and fewer unpleasant sequelæ, than by the use of alkaline sulphites.

These latter would seem, from our clinical observations and experiments thus far, to be more especially useful in retarding or arresting septic or necremic changes in the blood; and where the indications point simply to such ends, the sulphites are valuable agents. In the epidemic of erysipelas in this city, during the fall of 1863, in which many cases of an uncomplicated sthenic character occurred, they were found highly useful, and by their free exhibition, at an early stage of the disease, did much to cut short the period of its duration.

*Cardiac Disease—Post Mortem Appearance in.*—The death of the subject of the clinique, reported in the February number of the *Examiner*, (page 107), gave an opportunity for an examination of the pathological condition, which was duly presented to the class. Previous to his death, however, the attention of the class had been again called to him, and the hour pretty fully occupied in the physical exploration of his chest; so that on the examination of the lesion, after death, the students were enabled to appreciate the value of the pathognomanic symptoms they had become familiar with through life.

*Post Mortem:*—There was general serous infiltration of the areolar tissues, throughout the body; a moderate accumulation of serum in the peritonial and pleuritic cavities; and a completely œdematous condition of the left lung. The heart presented a rare and interesting combination of pathological changes. The mitral valve was thickened and indurated, and the opening through it moderately contracted. The tricuspid and semi-lunar valves were all natural in structure. The walls of the left ventricle were natural in thickness, but its cavity is moderately elongated and pouched at the apex. The walls of the right ventricle were greatly hypertrophied, being in some places more than three-quarters of an inch in thickness. The columnæ carnae were correspondingly increased in size, but the cavity of the ventricle was not materially altered in size or shape. The trunk and cardiac orifice of the pulmonary artery were so much dilated that the valves in the latter were insufficient to close it. The full explanation of these pathological changes in the heart, and their relation to the general symptoms, and physical signs presented before the death of the patient, was made very interesting and instructive to the clinical class.

*Early Diagnosis of Phthisis.*—P. McC., æt. 51, Irish; has been an inmate of the hospital some two or three weeks, suffering from general debility, and unusually enfeebled cardiac action. His general appearance, at the time of his admission, suggested a suspicion of crude tuberculosis as being at the origin of his troubles,—causing his debilitated condition, by impeding the functions of the lungs, diminishing the gross amount of assimilation, and causing an imperfect oxygenation of the blood, by which the vital properties of all the tissues were affected. Careful and repeated examinations, however, failed to detect any evidence of disease in the lung. Within the past few days a peculiarly fœtid odor, such as is occasionally observed accompanying the existence of an old glandular abscess, has been detected; and auscultation and percussion now exhibit such departure from the normal standards, as to warrant presenting him before the class, as an example of incipient tubercular phthisis. In the infra clavicular region of the right side, there is to be found a prolongation of the respiratory murmur, —not rough or loud, or in any other wise changed, but simply a prolonged expiratory sound. There is also a slight dulness on percussion in this region, though very slight and requiring close attention to detect the change. When the patient speaks, there is also a diffused vibratory jar perceptible. Thus there exist evidences, both of impaired capacity for air, and of increased density of structure in the upper part of that lung. In the cardiac region auscultation reveals feebleness of the heart's action, but without any abnormal sounds.

The primary cause of his trouble undoubtedly depends on a deficient innervation, causing a general feebleness of functional life; and the defective assimilation thus produced, is usually well adapted to the development of tubercular material, especially at his age.

The general indications of treatment in these early stages of the deposit and development of tubercular matter, are first to train the patient to regular muscular exercise, daily increased, and, as much as possible, out-doors in the open air; next, generous diet—let him eat all the food he can assimilate. If there be evidence of imperfect oxygenation of the blood, give six or eight grains of the chlorate of potassa in some mucilaginous fluid, after each meal. I am fully satisfied that this salt promotes oxygenation, and, consequently, assimilation. The presence of chlorine salts in the blood increases the capacity of that fluid for the absorption of oxygen from the air in the lungs. If in addition to this it be deemed advisable to produce an impression on the nervous system itself, the hyoscyamus and scutellaria will be found useful in allaying that nervous irritability which is almost universally manifested in a depressed condition of the system; while iron and nux vomica, or strychnia strongly increase nerve force and tone. The formula in general use in this hospital for such indications, is this:—

| | | |
|---|---|---|
| ℞ | Ext. Hyoscyamus,------------------- | 30 grs. |
| | Ext. Scutellaria,--------------------- | 30 grs. |
| | Ferri Sulphas,------------------------ | 30 grs. |
| | Aloes Pulv., ------------------------- | 10 grs. |
| | Ext. Nux Vom., ---------------------- | 10 grs. |

Mix, divide into 30 pills; take 1 before each meal.

For the nux vomica, strychnia may be substituted in the proportion of one-thirtieth of a grain to each pill; but the extract of nux vomica is preferable, if it can be procured of a reliable character, on account of its more persistent and uniform tonic action, while the alcaloid acts more directly as a stimulant to the spinal cord and medulla oblongata. Or the hydrastis canadensis, in the form of extract, and dose of one or two grains, may be substituted for the nux vomica, or be used in combination. In a proportion of these patients, and where tubercular deposit actually exists, substitute the cod liver oil for the pill, giving from a dessert to a table-spoonful three times a day. The oil may be emulsified by the addition of a half drachm or a drachm of the liquor potassæ to the ounce of oil, and in this way may be taken by patients of squeamish stomachs, or in cases of pyrosis or heartburn—producing an antacid effect, and should be taken from thirty to forty-five minutes after eating.

SERVICE—PROF. E. ANDREWS, M.D., SURGEON-IN-CHARGE.

*Radical Cure of Hydrocele.*—The occasional failure of the irritating injection to set up a sufficient amount of adhesive inflammation to effect a cure in this disease, and the objection to making more incisions than are absolutely necessary, rendering the seton obnoxious, has led Dr. Andrews to devise a modification, embracing the advantages of both plans without the inconveniences from the failure of the one, or the double incision of the other.

A. E. W. ætat. 23; admitted to hospital April 15th; hydrocele of the left side, caused by a kick in the scrotum by his sleeping bedfellow, about six months previously. The tumor had attained only a moderate size, but, owing to the existence of a varicocelous condition of the left spermatic cord, it was deemed desirable to get rid of the increased weight of the distended scrotum. The operation, as performed, consisted of the introduction of the canula in the ordinary manner, and the injection of about half an ounce of tincture of iodine, care being taken that the canula was within the sac itself, and not opening into the cellular tissue—(a neglect of which point had caused the sloughing away of the entire scrotum in a case which had fallen under the operator's notice, in which the timid surgeon had injected his irritating fluid into the tissue, instead of into the sac.) After the injection, a piece of tape was pushed up through the canula, by means of a probe, into the sac, and an inch or two of it left within in an irregular, crumpled-up ball; the canula was now withdrawn over the dependent end of the tape, leaving this last hanging out of the orifice. After from thirty-six to forty-eight hours, a sufficient amount of inflammation is excited, and the third or fourth day the tape may be withdrawn, freely bathed in pus.

By this means the amount of irritation can be pretty accurately graduated; and while it relieves the operation of the uncertainty of the simple injection, all the efficiency of the seton is obtained in a much neater and more workmanlike manner.

*Ununited Fracture of Humerus.*—Thomas McCarty; ætat.

23; Irish; fell off a load of lumber Sept. 25th, 1866, fracturing the right humerus, about the junction of the middle and upper thirds. Admitted to hospital March 14th, 1867; entire want of union between the two fragments; no apparent exciting cause. Was operated on by perforation soon after his admission, and strict attention given to his general health; no degree of inflammation, however, was induced, nor has there been yet, although the operation has been thrice repeated, making four operations in all in the space of thirty days, each operation increasing in severity in the extent and thoroughness with which the tissues have been broken up. Fifth week, from admission, operated again by introducing, through a small incision, a common inch and a half wood-screw through the fragments in such a way as to secure perfect apposition and immobility, except a slight lateral motion allowed by the play of the screw.

It is to be observed, that hitherto the operations have entirely failed in producing inflammation, and we may now look for some, though it is to be confessed, not a complete union as the result of this procedure.

April 22d, a sufficient degree of inflammatory action having been obtained, the screw was to-day withdrawn, and the arm dressed in the usual way.

*Andrews' New Plastic Operation.*—S. ætat. 21; in June, 1866, while carrying a coal-oil lamp broke the same, and, falling into the flames, was severely burned about the face; was admitted to hospital April 7th, 1867, with the loss of one eyebrow, ectropion of all the eyelids, quite extensive and much the worst of the left eye, and loss of both alæ of the nose. April 10th, operated on the left eye by the curved method with revolving flaps, as described in the EXAMINER for 1866, page 465. It is proposed to attempt the restoration of the alæ of the nose by the same method, and as the patient presents an unusually extensive field for the demonstration of the plastic operation, an effort will be made to furnish the readers of the EXAMINER with a full report of the various operations and their results.

